# Poorly differentiated clusters and tumor budding are important prognostic factors in colorectal carcinomas

**DOI:** 10.17305/bjbms.2021.6110

**Published:** 2021-09-03

**Authors:** Aura Jurescu, Alis Dema, Adrian Văduva, Adelina Gheju, Octavia Vița, Robert Barna, Codruța Lăzureanu, Marioara Cornianu, Sorina Tăban, Ciprian Duță, Stelian Pantea

**Affiliations:** 1 Department of Microscopic Morphology - Morphopathology, ANAPATMOL Research Center, “Victor Babeş” University of Medicine and Pharmacy, Timişoara, Romania; 2 Department of Pathology, “Pius Brînzeu” County Clinical Emergency Hospital, Timişoara, Romania; 3 Department of Surgery II, “Victor Babeş” University of Medicine and Pharmacy, Timişoara, Romania; 4 Department of Surgery II, “Pius Brînzeu” County Clinical Emergency Hospital, Timişoara, Romania; 5 Department of Surgery III, “Pius Brînzeu” County Clinical Emergency Hospital, Timişoara, Romania

**Keywords:** Colorectal carcinomas, tumor budding, poorly differentiated clusters, grading system, prognostic factors

## Abstract

The aim of our study was to assess the prognostic value of the two new grading systems based on the quantification of tumor budding - (GBd) and poorly differentiated clusters - (PDCs-G) in colorectal carcinomas (CRC). We performed a retrospective study on 71 CRC patients who underwent surgery at the Emergency County Hospital, Timişoara. CRC cases were classified based on hematoxylin-eosin slides, using the conventional grading system, GBd, and PDCs-G, respectively. We used two-tier and three-tier grading schemes for each system. Subsequently, we evaluated associations with other prognostic factors in CRC. Based on the three-tier GBd (GBd-3t) most cases (34/69, 49.27%) were classified as G3Bd-3t, while based on the conventional grading system, the majority of the cases (55/69, 79.71%) were considered G2. On the other hand, based on the three-tier PDCs-G system (PDCs-G-3t), most cases (31/69, 44.93%) were PDCs-G2-3t. We also noted a more significant association of GBd-3t with other prognostic parameters analyzed, as compared to the conventional grading system. Nodal status, tumor stage, and lymphovascular invasion were strongly correlated with GBd-3t (p=0.0001). Furthermore, we noted that PDCs-G-3t correlated more significantly than the conventional grading system with nodal status (p<0.0001), tumor stage (p=0.0003), lymphovascular invasion (p<0.0001), perineural invasion (p=0.005), and the tumor border configuration (p<0.0001). High GBd and PDCs-G grades correlate directly with other negative prognostic factors in CRC. Thus, these new parameters/classification methods could be used as additional tools for risk stratification in patients with CRC.

## INTRODUCTION

Globally and nationally, the incidence and mortality of colorectal cancer colorectal carcinomas (CRC) are increasing [1]. In men, it represents the third most common type of cancer, after lung and prostate cancer, while in women it ranks second place, after breast cancer [2].

CRC is a disease that has been extensively studied: Important progresses have been made with regard to diagnostic and treatment methods; prognostic and predictive factors, as well as some of their defects, have been identified. For example, there is a significant inter-observer variability regarding the degree of tumor differentiation (G), assessed using the grading system proposed by The World Health Organization (WHO grade) [1,3*-*5]. Moreover, using the TNM AJCC staging system (The TNM staging system - Tumor, Node, Metastasis proposed by The American Joint Committee on Cancer) [6] for CRC, an optimum classification of CRC patients with regard to recurrence and/or metastasis risk cannot be obtained [7]. Furthermore, it has been shown in the literature that clinical behavior may vary significantly in patients with the same stage of the disease, because the current staging classification offers limited prognostic information and is not able to anticipate the response to treatment [8].

In this context, a series of parameters that can be assessed on usual hematoxylin-eosin (HE) and immunohistochemical (IHC) stains are of interest lately, being more easily evaluated and more accessible than molecular markers, but most of them still need validation and standardization to be included in the histopathological report. Some of these new parameters, such as tumor budding (TB), poorly differentiated clusters (PDCs), tumor border configuration, and tumor infiltrating lymphocytes (TILs) could complete or even replace some classical prognostic factors for CRC [9].

Therefore, the aim of this study was to assess the prognostic value of TB and PDCs in CRC.

## MATERIALS AND METHODS

For this purpose, we conducted a retrospective study on a series of 71 CRC cases, out of which 21 CRC cases were previously diagnosed by endobiopsy and underwent robot-assisted surgery (da Vinci Xi^®^ Surgical System), between July 2015 and July 2016 in Surgery Clinic II of the “Pius Brînzeu” County Clinical Emergency Hospital from Timişoara and 50 consecutive CRC cases, diagnosed on resection specimens obtained in the Surgical Departments of “Pius Brînzeu” Hospital during the year 2014.

Criteria for exclusion from the study lot:

- Patients with different types of cancer, but carcinomas
- Patients who received neoadjuvant therapy before the surgical treatment
- Patients with tumor recurrences.

The CRC diagnosis was established after standard histopathological processing of the surgical resection specimens, within Pathological Anatomy Service of “Pius Brînzeu” County Clinical Emergency Hospital from Timişoara.

From the histopathological data of patients, we selected the following demographic and clinical-morphological parameters: Age of patients (<65 years/≥65 years); sex (F/M); localization of CRC (tumors of the right colon/left colon/rectum); histological type of the tumor: conventional adenocarcinomas (ADK NOS); and mucinous ADK – when the tumor component with extracellular secretion of mucin represents ≥50% of the tumor mass, according to the WHO criteria [4]; degree of tumor differentiation using three grading systems described below; and the depth of tumor invasion into the intestinal wall: Early invasive (pT1**-**pT2) or deeply invasive (pT3**-**pT4) tumors; lymph node status – absence (pN-) or presence of lymph node invasion (pN+); presence of distant metastases – pathologically documented (pM1); TNM AJCC stage, according to AJCC Staging Manual, 8^th^ edition [10]; absence/presence of lymphovascular invasion (LVI−/LVI+), TILs−/TILs+; tumor necrosis (absent/present); tumor ulceration (absent/present); and tumor border configuration (pushing/infiltrating type).

From the group of 71 CRC cases, two were diagnosed as mucinous ADK. Based on the fact that mucinous ADK are not graded using the percentage of glandular structure formation (according to the WHO classification of digestive system tumors, 4^th^ edition) [4] and considering the absence of a consensus about the evaluation method of TB or PDCs in the presence of mucin, we did not grade the two cases of mucinous ADK by any of the three grading systems used in this study (the WHO grade, GBd or PDCs-G). Therefore, TB and PDCs quantifications were applied only for the 69 cases of ADK NOS.

Next, we classified the tumors using the new grading systems based on the evaluation of TB (GBd) and PDCs (PDCs-G) on HE stained slides and we analyzed the relationship between these parameters and other prognostic factors (classical and more recently described) in CRC. Furthermore, we compared the two grading systems based on the quantification of TB (GBd) and PDCs (PDCs-G) with the WHO grade, as well as GBd and PDCs-G between themselves, in order to assess the prognostic value of these new parameters in CRC.

### Evaluation of the WHO grade

**FIGURE 1:**
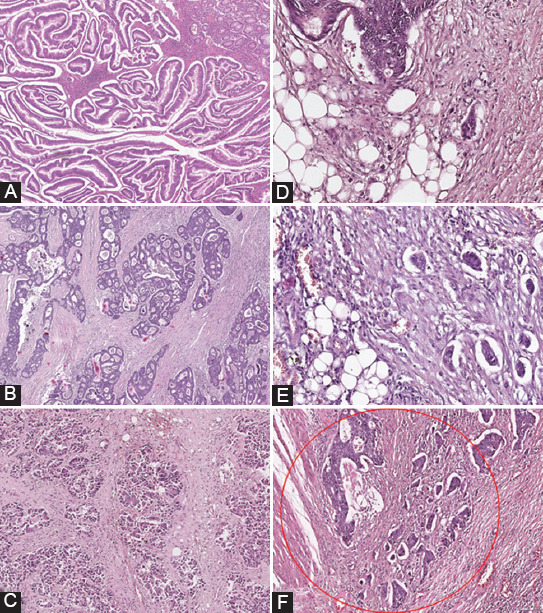
Comparative sample images between the WHO grade and PDCs-G in CRC: (A) WHO Grade 1 CRC, HE× 100; (B) WHO Grade 2 CRC, HE× 100 (C) WHO Grade 3 CRC, HE× 100 (D) PDCs - G1 CRC, HE× 400 (E) PDCs - G2 CRC, HE× 400 (F) PDCs - G3 CRC, HE× 100. WHO grade: The conventional grading system; PDCs-G: The grading system based on the quantification of poorly differentiated clusters; CRC: Colorectal carcinomas.

The degree of tumor differentiation was assessed for ADK NOS according to the WHO criteria [4], based on the percentage of glandular formation (Figure 1), as follows: G1 – well differentiated tumors (>95% glandular formation), G2 – moderately differentiated tumors (formation of glands in 50% - 95% of the tumor), G3 – poorly differentiated tumors (glandular formation in 0 - 49% of the tumor), and G4 – undifferentiated carcinomas (no gland formation detected, no mucin present, squamous, or neuroendocrine differentiation). Moreover, we distributed the ADK NOS cases into two groups of differentiation degrees, depending on the percentage of glandular formation (≥ 50% and <50%): Tumors with low grade (G1 - G2) and high grade (G3 – G4) of malignity [1,3,4]

### Evaluation of GBd grade

TB is defined as a single tumor cell or a cell group of up to four tumor cells (1–4 tumor cells) that did not represent areas of glandular disruption produced by the inflammatory infiltrate [5,11,12]. We evaluated TB for all of the cases included in the study on the invasive front of the tumor, according to the International TB Consensus Conference (ITBCC) recommendation [11].

**FIGURE 2:**
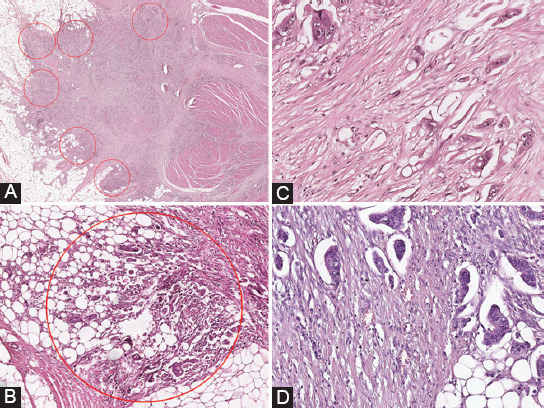
CRC sample images showing the evaluation of TB at the tumor invasion front: (A) a preliminary evaluation of the whole front of tumor invasion at low magnification, pT3 CRC, HEx40; (B) the field with the highest TB density in a circular area with a diameter of 0.785 mm2 at the tumor invasion front - the hotspot, G3Bd, HEx200; (C, D): TB - isolated cells or groups of ≤4 tumor cells and PDCs - groups of ≥5 tumor cells, without gland formation, HEx400; CRC: Colorectal carcinoma; TB: Tumor budding; G3Bd: High grade tumor budding (with ≥10 TB) in the grading system based on TB quantification; PDCs: Poorly differentiated clusters.

The first step in TB assessment was the examination of all HE stained slides from every respective case, in view of selecting the slides and paraffin blocks to be used in the study. For each case, after evaluation under the optical microscope, we selected a slide with the tumor section that contained the highest number of TB at the front of tumor invasion, from the area with maximal tumor infiltration into the intestinal wall. After a preliminary evaluation of the whole front of invasion under the optical microscope, at low magnification (×40), we selected for evaluation the field with the highest TB density. This was considered a *hotspot area* and underwent subsequent evaluation at an intermediary magnification (×200), the size of the field being 0.785 mm^2^, Figure 2. Depending on the TB number identified in the hotspot area, from the front of tumor invasion, the cases of CRC were grouped in three categories - GBd-three-tier (*GBd-3t*): Tumors with 0–4 TB were considered G1Bd-3t, those with 5–9 TB were included in the G2Bd-3t group, while cases with TB ≥10 were G3Bd-3t, according to the recommendations of the ITBCC of 2016 [11].

Further, we classified the tumors in a two-tier system – GBd-two-tier (*GBd-2t*): low GBd (G1Bd-2t) for tumors with TB ≤9 and high GBd (G2Bd-2t) for tumors with TB ≥10, according to the model used by Graham et al. and adopted by other authors [13-15].

After classifying the tumors using the GBd, we compared the distribution of cases depending on the two grading systems: GBd based on TB quantification and classical WHO grade. Then, we analyzed the associations between GBd (GBd with three-tier: GBd-3t and two-tier: GBd-2t, respectively) and all the other prognostic parameters investigated, in order to verify whether the classification system based on TB quantification is a promising prognostic factor in CRC evaluation.

### Evaluation of PDCs-G grade

According to Ueno’s definition, PDCs are groups of ≥5 cancer cells that are not arranged in a glandular structure and can be observed in the area with their highest density (hotspot), at the front of tumor invasion [5,16].

We graded ADK NOS depending on the presence of PDCs at the front of tumor invasion, on HE stained slides, using the method proposed by Ueno et al. in 2012 [5]. In this regard, we selected for each case the block that included the area of invasion, with the maximal tumor infiltration into the intestinal wall. Initially, the slides were examined under the optical microscope at low magnification (×40) to identify the area with the highest density of PDCs along the tumor invasion front. This area was considered “hotspot” and was subsequently evaluated at an intermediary magnification (×200), where the field size was 0.785 mm^2^. The quantification system we used for PDCs was similar to the one for TB, with three grades, corresponding to <5, 5–9 and ≥10 PDCs, respectively. Depending on the number of PDCs identified in the assessed area, we classified CRC cases in three categories of PDCs grade (PDCs-G-3t): tumors with <5 PDCs were considered PDCs-G1-3t, those with 5-9 PDCs were allocated in the PDCs-G2-3t category, while cases with ≥10 PDCs were classified as PDCs-G3-3t, Figure 1 [5]. After that, we graded the tumors using a system with two-tier PDCs (PDCs-G-2t), establishing a cutoff value of 10 PDCs, similar to the TB quantification method described above.

We performed a comparative analysis of the distribution of cases according to the two grading systems: PDCs-G and WHO grade, to evaluate if the new grading system – PDCs-G can be used as a promising prognostic parameter for patients with CRC.

In the end, we established which of the newly proposed grading systems correlates significantly with classical prognostic factors.

### Ethical statement

All the procedures included in this study were carried out according to the principles of the Declaration of Helsinki of good clinical practice. Each patient signed an informed consent form, allowing the use of the tissue fragments in scientific studies.

### Statistical analysis

The parameters we gathered were statistically analyzed using the functions of Microsoft Excel and Graph Pad Prism v6 software. We used the Chi-square and Fisher’s exact tests to analyze the correlations and/or the differences between clinical-pathological factors. The p value was considered as statistically significant when it was lower than 0.05.

## RESULTS

The group of patients that we studied was made up of 71 cases of CRC: 69 were ADK NOS/conventional type and two cases (3%) were mucinous ADK.

The statistical analysis was carried out for the 69 cases of ADK NOS, the demographical and clinical-morphological characteristics being presented in Tables 1-3.

**TABLE 1:**
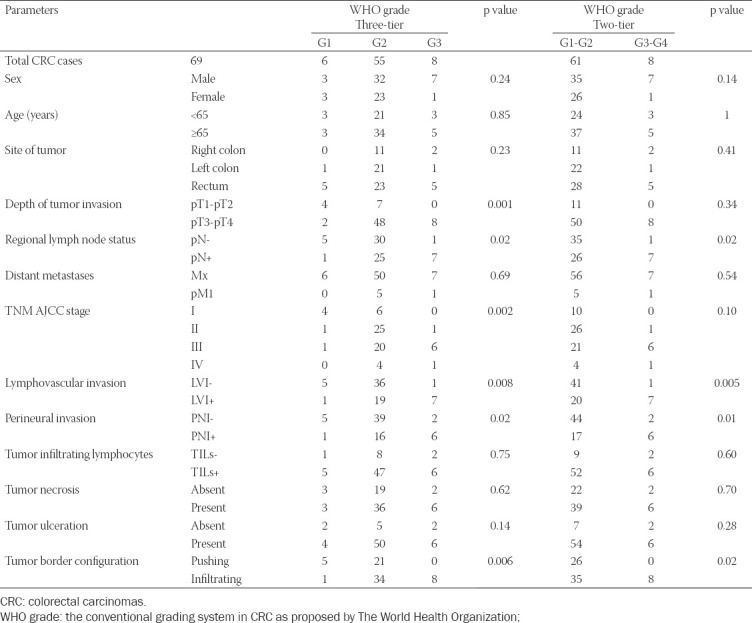
The distribution of CRC cases according to the two WHO grading systems and statistical correlations with prognostic parameters, analyzed with the Chi-square test, and Fisher’s exact test.

**TABLE 2:**
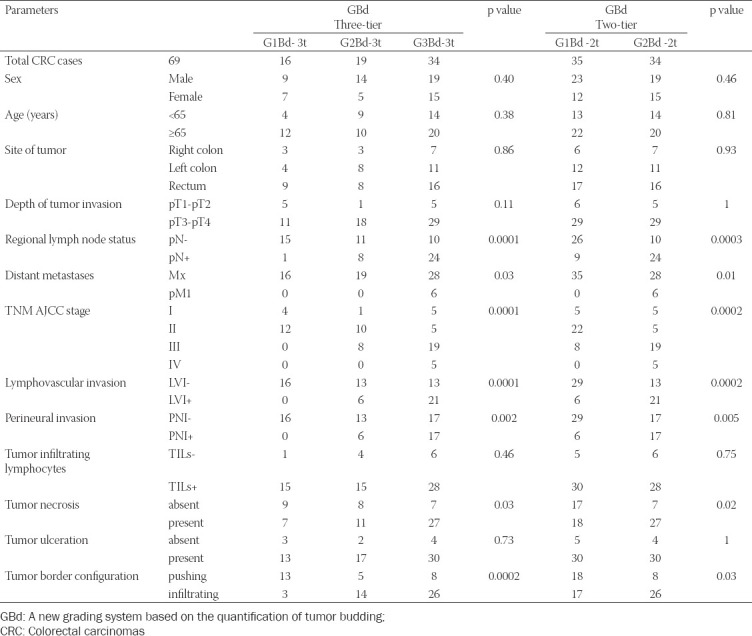
Statistical analysis of the correlations between the two GBd grading systems and the prognostic parameters in the CRC, using the Chi-square test, and Fisher’s exact test

**TABLE 3:**
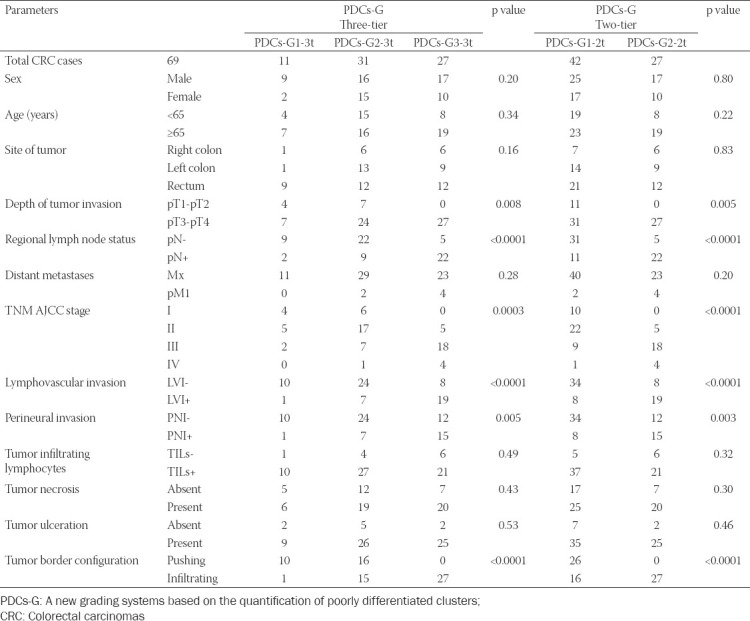
Statistical analysis of the correlations between the two PDCs-G grading systems and the others prognostic parameters in the CRC, analyzed with the Chi-square test and Fisher’s exact test

**FIGURE S1:**
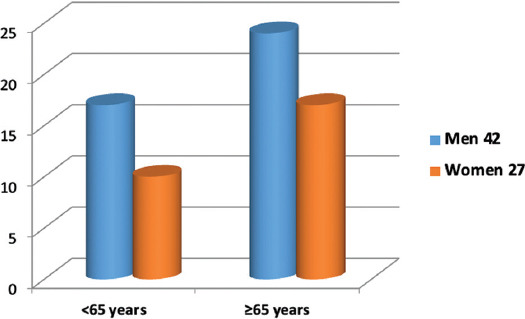
Distribution of colorectal carcinoma cases according to patients’ sex and age.

**FIGURE S2:**
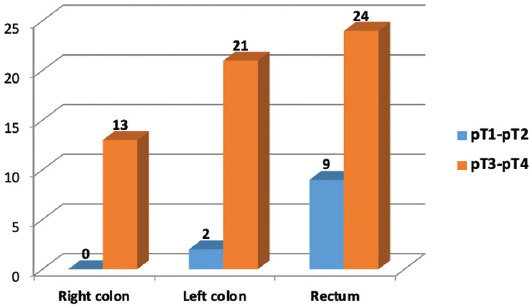
Distribution of colorectal carcinoma cases according to the depth of the tumor invasion and the location of the tumor in the colon.

We identified 42 (60.87%) male patients and 27 (39.13%) women (Figure S1). Twenty-seven patients (39.13%) had ages <65 years and 42 patients (60.87%) were over 65 years of age (Figure S1). Regarding the localization of CRC, 13 cases (18.84%) were found in the right colon, 23 cases (33.33%) in the left colon, and 33 cases (47.83%) in the rectum (Figure S2). According to the depth of tumor invasion into the intestinal wall, the cases were assigned as follows: 11 cases (15.94%) were tumors presented early invasion (pT1-pT2) and 58 cases (84.06%) invaded deeply into the intestinal wall (pT3-pT4) (Figure S2). A total of 33 (47.83%) patients presented metastases into the regional lymph nodes (pN+). Six patients (8.7%) had distant metastases (pM1). The cases we studied were classified according to the TNM AJCC staging system as follows: 10 cases (14.49%) were Stage I, 27 cases (39.13%) Stage II, 27 cases (39.13%) Stage III, and 5 cases (7.25%) Stage IV. Twenty-seven cases (39.13%) presented LVI+, in 23 cases (33.33%), we noted perineural invasion (PNI) and 58 cases (84.06%) presented TILs+. Tumor ulceration was identified in 60 cases (86.96%). Tumor necrosis was present in 45 cases (65.22%). Regarding the configuration of the invasion front, we observed that 26 cases (37.68%) presented the pushing type and 43 cases (62.32%) had infiltrating type.

Using the classical WHO grade, we noted that most of our cases - 55/69 (79.71%) were G2, followed by 8/69 (11.59%) G3 and 6/69 (8.7%) G1. None of our cases was graded as G4. Considering the two-tier system: low-grade malignity (G1-G2) and high-grade malignity carcinomas (G3-G4), 61 cases (88.41%) were G1-G2 tumors, and 8 cases (11.59%) were G3-G4.

Based on the GBd-3t grading system, we observed the following distribution of cases: 16 cases (23.19%) G1Bd-3t, 19 cases (27.54%) G2Bd-3t, and 34 cases (49.27%) were G3Bd-3t. Using the GBd-2t system, 35 cases (50.73%) were G1Bd-2t and 34 cases (49.27%) were G2Bd-2t.

According to the PDCs-G-3t grading system, we obtained the following distribution of cases: 11 cases (15.94%) PDCs - G1-3t, 31 cases (44.93%) PDCs - G2-3t, and 27 (39.13%) PDCs - G3-3t cases. After using the PDCs-G-2t system, we distributed the patients as follows: 42 (60.87%) cases were PDCs-G1-2t and 27 were (39.13%) PDCs - G2-2t cases.

**FIGURE S3:**
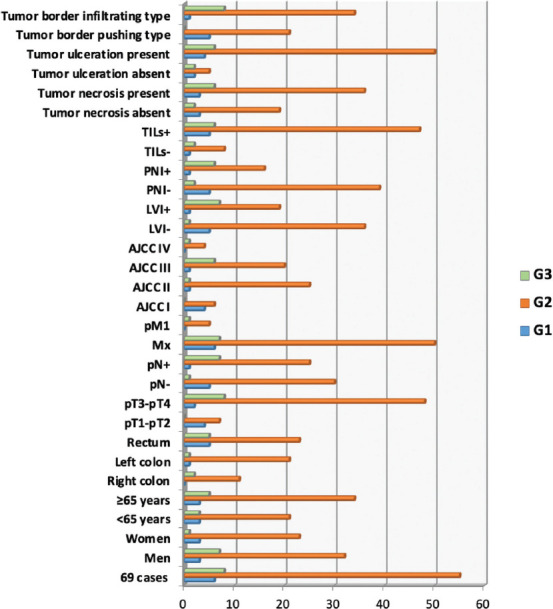
The correlation between clinicopathological parameters and the WHO grade.

The assessment of associations between WHO grade and other classical prognostic factors (Table 1, Figure S3) showed positive correlations between the three-tier WHO system and tumor extension into the intestinal wall - pT1-pT2/pT3-pT4 (p=0.001), lymph node status – pN (p=0.02), TNM AJCC tumor stage (p=0.002), LVI (p=0.008), PNI (p=0.02), and the tumor border configuration (p=0.006). After assigning cases according to the two-tier WHO system and analyzing its relationship with the other prognostic factors, we observed direct correlations between WHO grade and pN (p=0.02), LVI (p=0.005), PNI (p=0.01) and the tumor border configuration (p=0.02), Table 1.

**FIGURE S4:**
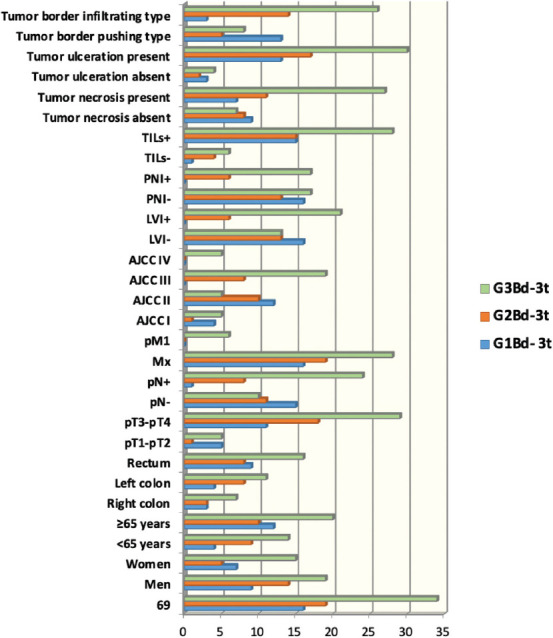
The correlation between clinicopathological parameters and GBd grade.

Statistical analysis of the associations between the prognostic parameters and the GBd-3t grade showed the positive correlations between high grade GBd and pN+ (p=0.0001), pathologically documented distant metastases (pM1, p=0.03), advanced TNM AJCC stage (p=0.0001), LVI+ (p=0.0001), PNI+ (p=0.002), presence of necrosis (p=0.03), and the infiltrative configuration of tumor invasion front (p=0.0002), Table 2, Figure S4. Regarding the analysis of the relationship between GBd-2t system and prognostic parameters, we observed a direct relation between G2Bd-2t and pN+ (p=0.0003), pM1 (p=0.01), advanced TNM AJCC stage (p=0.0002), LVI+ (p=0.0002), PNI+ (p=0.005), presence of necrosis (p=0.02), and infiltrative configuration of the invasion front (p=0.03), Table 2.

**FIGURE S5:**
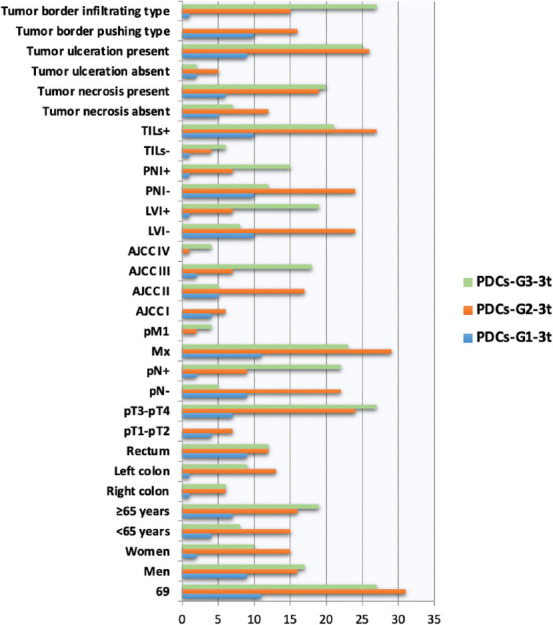
The correlation between clinicopathological parameters and poorly differentiated clusters grade.

As it is shown in Table 3, we found statistically significant associations (positive correlations) of PDCs-G-3t with pT1-pT2/pT3-pT4 (p=0.008), pN (p<0.0001), TNM AJCC stage (p=0.0003), LVI (p<0.0001), PNI (p=0.005), and the tumor border configuration (p<0.0001), Figure S5. With the help of the PDCs-G-2t system, statistically significant correlations were obtained, more important or identical to PDCs-G-3t, with: pT1-pT2/pT3-pT4 (p=0.004), pN (p<0.0001), TNM AJCC stage (p<0.0001), LVI (p<0.0001), PNI (p=0.003), and tumor border configuration (p<0.0001), as shown in Table 3.

## DISCUSSION

Tumor stage, as assessed using the TNM AJCC system, remains the most important prognostic parameter for the classification of patients with CRC into groups that can benefit from different types of treatment [17,18]. However, according to some authors, the TNM AJCC stage is not a powerful enough predictor for the post-surgical outcome in patients with CRC [19,20].

On the other hand, the histological grade of colorectal ADK is constantly reported and is recognized as one of the most important parameters correlated with CRC aggressiveness [4]. Nevertheless, at present, a consensus regarding CRC grade was not reached, different grading systems classifying colorectal ADK in two, three or four categories [8,21]. These systems take into consideration tumor architecture/gland formation, cytological criteria, and one or more microscopic features, evaluation methods varying largely in general practice [3,22,23].

The most frequently used grading method for CRC is based on the percentage quantification of glandular or tubular structure formation (the architectural type) [1], using the classification system with four- tier [4]. To eliminate the difficulties encountered with three- or four-tier systems, the WHO recommended using a binary grading system that recognized the low grade CRC category, where >50% of the tumor presents glandular formation (corresponding to Grades 1 and 2 from the four- tier classification) and the high grade CRC category, when <50% of the lesion presents tubular structures (Grades 3 and 4 from the four- tier classification) [1,3,4]. In the 5^th^ edition, the WHO recommends using a system with two grade classes for the classification of CRC [1]. This revised classification is based on arguments that show similarities in the evolution of patients with well and moderately differentiated ADK [24].

Grading of heterogeneous tumors is another source of variability in the CRC classification, which is largely attributed to the imprecise and subjective nature of the evaluation [5,8,21-23]. In many reference guidelines, there are discrepancies regarding the area that should be classified in the CRC: The area with the least differentiated tumor component or the predominant grade. Internationally, guidance on the grading of heterogeneous CRC tumors are directed toward the use of the worst, rather than the predominant grade [1,3,4,6,10,24]; however, the extension of the area to be analyzed is not specified, thus increasing the amount of observer bias [21-23].

Moreover, the WHO grade could be applied only to NOS adenocarcinoma, but not to other histological types. Again, it appears that the two-tier WHO classification suffers from several limitations and a decline in the prognostic importance of this parameter has been observed [5,8,22,23].

Given the above-mentioned controversies, criteria for CRC grading needed to be refined and perfected. Although the analysis of certain molecular markers seems useful and attractive from the perspective of prognostic and predictive value in patients with CRC [1,25], histopathological and IHC investigations, by far more easily done and cheaper, could offer solutions for a better classification of patients with CRC. In this regard, TB [16,26], PDCs [27,28], and local immune response [29] were carefully studied as parameters with promising results for the improvement of treatment management in patients with CRC.

Opinions about reporting TB in CRC are divided among authors. Until some universally accepted criteria are established, the rules discussed at the Consensus Conference on TB – ITBCC of 2016 should be applied. These are the most used criteria in this domain and are easy to apply; by implementing them widely, the assessment of CRC would be improved in daily practice. Another indicator considered to be a promising prognostic factor in CRC is PDCs. Both TB and PDCs are considered to be the histological and morphological expression of epithelial-mesenchymal transition (EMT); based on their similar morphology, the simultaneous presence of TB and PDCs, as well as the presence of EMT in both situations, it has been suggested that PDCs may represent the result of sequential transformation of TB [5,9,30 - 33].

In our study, we classified cases according to both WHO grading systems (with four and two grade classes, respectively). However, after reclassifying cases based on the binary WHO system, we did not obtain more statistically significant correlations between the binary WHO system and the analyzed prognostic factors, except for LVI (p=0.005) and PNI (p=0.01). In the literature, a significant variation of inter-observer concordance was also observed regarding the binary grading system; therefore, the WHO grading system based on the percentage quantification of tubular/glandular structures has limited prognostic value [ 34].

Therefore, we evaluated TB and PDCs in 69 cases of ADK NOS, on slides stained with HE, under the optical microscope, on a field of 0.785 mm^2^, corresponding to a × 20 objective, following the recommendations of ITBCC. Given the variability of field dimensions depending on the microscope used, to standardize the evaluation of TB, it was elaborated a conversion scale, depending on the type of microscope used, up to the area of 0.785 mm^2^ [11]. Similarly, in their guides for the treatment of CRC devised in 2014 [ 35], Japanese Society for Cancer of the Colon and Rectum recommends the assessment of a single microscopic field from tumor invasion front which contains the highest number of TB (the hotspot method), under a × 20 objective, on HE stained slides [ 35].

In our study, after carrying out a comparative analysis between the GBd-3t grading system and the WHO grade, we observed a different distribution of CRC cases: using the GBd-3t grading system, most cases (49.27%) were classified as G3Bd-3t, while based on the WHO grade; and most cases (79.71%) were considered G2.

In addition, we noted more significant associations of GBd-3t with the other prognostic parameters analyzed, as compared to the WHO grade. Thus, pN status, TNM AJCC stage, and LVI correlate very strongly with GBd-3t, in all situations the value of p being 0.0001. Statistical analysis of associations between the other clinical and pathological parameters that we studied and GBd-3t grade showed more important correlations than with WHO grade and other parameters, like: The configuration of the tumor invasion front (p=0.0002), PNI (0.002), and pM (p=0.03) and the presence of tumor necrosis (p=0.03). Moreover, with the help of the GBd-2t system, we obtained more important correlations than with GBd-3t system for pM (p=0.01) and tumor necrosis (p=0.02). After using both grading systems, GBd-3t and GBd-2t, for the rest of the prognostic parameters investigated, the values we obtained for p were very similar.

To define GBd-2t, we chose the cut off value of ≥10 TB and we believe that using this binary system would better serve TB evaluation in CRC. Consistent with our results, several authors showed that ≥10 TB on one microscopic field of x20 would be the optimum cut-off value for defining high grade TB [13, 36]. In their study, Mitrovic et al. [ 37] argued that the value of TB >10 would be adequate for high grade TB. Furthermore, Graham et al. demonstrated that the presence of TB has a negative effect on the survival of patients with CRC, showing that the relative risk of death by disease is 2 –3 times higher when high grade TB (≥10 TB) is present [13].

In our study, high grade TB proved to be a factor that correlates strongly with pN+. Our data confirm other studies, which support the idea that high grade TB correlates strongly with the other negative prognostic factors: A significant association between the presence of lymph node metastases with LVI and high grade TB has been shown [ 38]. Furthermore, in a study made by Hase et al., patients with moderate and high grade TB presented significantly higher local recurrence rates and a significant decrease of 5- and 10-year survival rates [ 39].

TB, as a marker of tumor aggressiveness, was associated with the evolution of patients with CRC, high grade TB being correlated with a negative prognosis [ 31, 32, 40]. Some studies suggest that TB can have a more important prognostic value than histological grade, independent of the tumor border configuration [ 31]. The guides elaborated by the European Society of Medical Oncology in 2012 recognize TB as a potential prognostic factor for incipient CRC [ 41]. Most research show that the presence of a high number of TB has a negative impact on prognosis, by correlating with LVI, pN and M [ 37], and even being considered as an independent prognostic marker in CRC with N0 [13]. TB is predictive for pN+ on resection pieces of superficial T1 and T2 rectal cancers, suggesting that it can be useful as a prognostic indicator in patients with recurrence risk after local excision of the tumor [ 31]. Compton [ 42] states that TB represents an additional prognostic marker, together with histological grade, PNI, and the tumor border configuration. More recently, high grade TB is considered an adverse prognostic factor, often independent from the pathological stage of the disease [26, 42]. Hence, quantifying and reporting TB are justified, as is classifying patients according to TB grades.

Regarding therapy, the presence of TB on preoperative biopsies of CRCs would impose applying neoadjuvant treatment [11]. According to some authors, it is extremely important to report TB in the case of malignant polyps and T1 tumors because the presence of high grade TB increases the risk of developing lymph node metastases by 15 –20% [ 43]; thus, TB represents a predictor of lymph node metastases, entailing segmentary resection with regional lymphadenectomy. If patients with Stage II CRCs would benefit from improved survival after chemotherapy, assessing TB would have an important role in the treatment algorithm [11, 37].

Although, practically, IHC use for TB quantification in all CRC cases is impractical, in certain situations it may be necessary, especially in the presence of a prominent peritumoral inflammatory reaction that makes assessment difficult/impossible. Thus, when there is a dense inflammatory infiltrate or marked desmoplasia in the peritumoral stroma, hiding tumor cells, TB is difficult to identify on HE-stained slides [27,30, 31]. To correctly identify TB, using IHC (cytokeratin) staining is advised, when there is glandular fragmentation associated with inflammatory infiltrate, in the presence of mucin areas or in the case of artifactual retractions of adjacent stroma [30, 36, 44]. However, criteria have to be established and a consensus needs to be reached for the IHC assessment of TB, while the gastrointestinal pathology community has the role to introduce TB evaluation in daily practice and to eliminate barriers that stand in the way of its generalized report [ 37].

Due to their sizes, PDCs are easier to identify on usual stains; additional IHC techniques are not necessary for their distinction, making their evaluation cheaper and easier [5,28,30, 45, 46].

Although there are few studies that compare directly PDCs –G with the WHO grade, the grading outline for CRC based on the quantification of PDCs seems to display a better inter-observer reproducibility as compared to the WHO grade and better prognostic and predictive values [ 45]. In our study, after reclassifying cases based on the new grading system (PDCs -G), we observed a more uniform distribution of cases: About 16% - PDCs-G1, 45% - PDCs-G2, and 39% - PDCs-G3. After analyzing the associations between classical prognostic parameters and grading systems based of PDCs quantification, we noted that PDCs-G-3t correlated more significantly than the WHO grade with pN, TNM AJCC stage, LVI, PNI, and tumor border configuration. After classifying cases using PDCs-G-2t, correlations with all analyzed prognostic factors were even more important; moreover, the most significant statistical associations were obtained between this grading system (based on the quantification of PDCs, a system with two grades – PDCs-2t), and pN, TNM AJCC stage, LVI, and tumor border configuration. From the perspective of comparing PDCs-G system with GBd, we observed that with PDCs-G, we obtained more significant correlations for tumor extension into the intestinal wall (pT), TNM AJCC system, LVI, and the tumor border configuration; instead, using GBd we reached more statistically significant results for pM and tumor necrosis.

Using this new grading system for patients with CRC, Bonetti Barresi et al. [28, 46-50] confirm and strengthen the idea that PDCs-G guarantees a more objective interpretation, being a more valuable prognostic factor than the classical system based on histological grading (WHO grade) and even than TNM AJCC stage. In their studies, Barresi et al. emphasize the fact that high grade PDCs represent an adverse prognostic factor in CRC [46, 48, 49]. The correlation between PDCs and lymph node metastases and micrometastases is more important for tumors with high grade PDCs [ 33, 45, 47, 50, 51]. According to other studies, PDCs-G proved to be an important parameter for determining the risk of pN+ in CRCs with incipient invasion into the intestinal wall (pT1) [ 33, 34]. To date, only a few published studies quantified PDCs on preoperative biopsies [ 47, 50], but it was demonstrated that the presence of high grade PDCs is correlated with an infiltrative tumor border configuration, LVI+ and high grade TB [ 50]. The number of PDCs on biopsies could be underestimated because tissue fragments are often collected from the superficial part of the tumor that does not include the invasion front where PDCs are generally more numerous [ 47, 50]. The agreement between pathologists on PDCs evaluation on biopsies is higher than with WHO grade [ 50]. However, on preoperative biopsies, interpretation difficulties for PDCs can be encountered, in the presence of necrosis, inflammation, ulceration, tissue fragmentation, tangentially cut glands, thermal-induced artifacts, or the superficiality of sampling that does not include the invasion front [30, 33]. Moreover, according to data from literature, PDCs-G promises to be a more credible and precise instrument for the prediction of metastatic potential of CRC, as compared to WHO grade, LVI, or the tumor border configuration [5, 45, 46, 49].

This new classifying system offers prognostic information, as well as a lot of other advantages concerning reproducibility, but because it is a relatively new parameter and the number of published studies is low, its evaluation implies some unresolved issues, needing further validation and standardization [5,28, 45, 48].

Thus, while some studies showed that the PDCs classifying system proposed by Ueno offers a good assessment of the risk for patients with CRC, optimum cut offs for each PDCs grade, and the quantification method of PDCs in special histological types were not exactly established. An unresolved issue remains the evaluation of PDCs in mucinous ADKs. According to Barresi et al., PDCs-G could be assessed in mucinous ADK only if identified in areas with minimum extracellular mucin at the tumor invasion front [ 49, 52]. Grading mucinous ADK based on PDCs-G is problematic given the fact that the paper that initially proposed this grading system does not include a clear definition of clusters in mucinous areas [5]. Large studies using multivariate analysis, well-characterized patient series and reproducible methods are still necessary to determine optimum cut-off values, both on surgically removed pieces for the evaluation of PDCs and on biopsies for the accurate classifying of risk in patients with CRC.

However, despite the existence of studies that confirm the value of PDCs in CRC, no meeting was schedule to establish a consensus about the evaluation and reporting method for PDCs. The introduction of TB and PDCs in the histopathological report of CRC is delayed because there is a great variability in their evaluation and reporting. Larger cohort studies are necessary to confirm the prognostic value and the significance of TB and PDCs, both in patients with colon cancer and rectum cancer, especially for subgroups with radiotherapy treatment. However, TB and PDCs are parameters that need to be known and the pathologist needs to be familiar with their identification and quantification, if standardization of their assessment and report will lead to the implementation of these parameters with prognostic and predictive value in the current diagnostic of CRC. Consequently, TB and PDCs should be taken into consideration during multidisciplinary meetings to facilitate therapeutic decisions in daily clinical practice.

### Study Limitations

The study was performed on a reduced number of cases; a greater number of cases would bring confir mation of our results. Another drawback of our study is the absence of clinical follow up data that would favor the identification of possible correlations with short-and long-term survival outcomes.

## CONCLUSION

High-grade TB and PDCs are parameters that are associated with adverse prognostic factors in CRC, independent of the binary/three-tier systems used in our study. We consider that the binary grading systems could be more useful for the classification of CRC cases due to the rapidity in the evaluation and, implicitly, in the applicability in the daily practice.

Both TB and PDCs correlate with the same unfavorable prognostic factors, but given that PDCs are easier to assess as compared to TB, on usually stained slides, PDCs appear to be a more reproducible parameter than TB for routine evaluation of CRC.

Considering the prognostic relationship between PDCs and TB, we suggest the standardization of the assessment method for PDCs and TB on HE and IHC stains, for the special histological types of CRC, as well as the inclusion of TB and PDCs in the histopathological report, with the specification of the number of TB and PDCs and the corresponding grade category (GBd and PDCs-G, respectively).

The biological relationship between TB and PDCs, the uniformity and reproducibility in the assessment and reporting of these parameters, the establishment of cumulative schemes for quantifying several parameters at the tumor invasion front, the role of immunohistochemistry and automated detection algorithms in TB and PDCs quantification represent new arid directions to be explored in CRC.
